# Chemokine Receptors and Phagocyte Biology in Zebrafish

**DOI:** 10.3389/fimmu.2020.00325

**Published:** 2020-02-25

**Authors:** Frida Sommer, Vincenzo Torraca, Annemarie H. Meijer

**Affiliations:** Institute of Biology Leiden, Leiden University, Leiden, Netherlands

**Keywords:** *Mycobacterium marinum*, infection, wounding, zebrafish, cancer, inflammation, chemokine receptor, phagocytes

## Abstract

Phagocytes are highly motile immune cells that ingest and clear microbial invaders, harmful substances, and dying cells. Their function is critically dependent on the expression of chemokine receptors, a class of G-protein-coupled receptors (GPCRs). Chemokine receptors coordinate the recruitment of phagocytes and other immune cells to sites of infection and damage, modulate inflammatory and wound healing responses, and direct cell differentiation, proliferation, and polarization. Besides, a structurally diverse group of atypical chemokine receptors (ACKRs) are unable to signal in G-protein-dependent fashion themselves but can shape chemokine gradients by fine-tuning the activity of conventional chemokine receptors. The optically transparent zebrafish embryos and larvae provide a powerful *in vivo* system to visualize phagocytes during development and study them as key elements of the immune response in real-time. In this review, we discuss how the zebrafish model has furthered our understanding of the role of two main classes of chemokine receptors, the CC and CXC subtypes, in phagocyte biology. We address the roles of the receptors in the migratory properties of phagocytes in zebrafish models for cancer, infectious disease, and inflammation. We illustrate how studies in zebrafish enable visualizing the contribution of chemokine receptors and ACKRs in shaping self-generated chemokine gradients of migrating cells. Taking the functional antagonism between two paralogs of the CXCR3 family as an example, we discuss how the duplication of chemokine receptor genes in zebrafish poses challenges, but also provides opportunities to study sub-functionalization or loss-of-function events. We emphasize how the zebrafish model has been instrumental to prove that the major determinant for the functional outcome of a chemokine receptor-ligand interaction is the cell-type expressing the receptor. Finally, we highlight relevant homologies and analogies between mammalian and zebrafish phagocyte function and discuss the potential of zebrafish models to further advance our understanding of chemokine receptors in innate immunity and disease.

## Introduction

Phagocytosis refers to the recognition and internalization of particles larger than 0.5 μm into a plasma membrane-derived vesicle called the phagosome. Phagocytes are cells that can phagocytose harmful particles, pathogens, and dying cell debris. Phagocytes are broadly divided into professional and non-professional phagocytes (1). In non-professional phagocytes like epithelial cells, endothelial cells, and fibroblasts, phagocytosis is a facultative function as these cells have other tissue-resident functions, although they can contribute to tissue homeostasis by phagocytosing apoptotic debris (2). In contrast, professional phagocytes efficiently identify, engulf, and clear invading pathogens, harmful substances, and dying cells. This group includes highly motile cells such as neutrophils, monocytes, macrophages, eosinophils, mast cells, and dendritic cells as well as tissue-resident cells like osteoclasts (3). Professional phagocytes express multiple specialized membrane-bound receptors that recognize target particles of different nature. Pattern recognition-receptors (PRRs) identify pathogen-associated molecular patterns (PAMPS) and damage-associated molecular patterns (DAMPS) and activate the immune response (1, 3). The phagocytosis process, itself is initiated by other surface receptors. Among these, scavenger receptors mediate the phagocytosis of endogenous ligands, like lipoproteins, as well as microbial invaders. Opsonic receptors recognize targets detected and bound by soluble host molecules, such as complement proteins and antibodies. Receptors for apoptotic cells recognize soluble cues secreted by dying cells (e.g., lysophosphatidylcholine and ATP) or characteristic molecules exposed on the surface of dying cells, such as phosphatidylserine (1, 2). Professional phagocytes play pivotal roles in immunomodulation, development, pathogen clearance and antigen presentation (2, 3).

In addition to pattern recognition and phagocytic receptors, phagocytes express various types of chemokine receptors that coordinate cell movement and confer certain functional properties to these cells (4, 5). Chemokine receptors belong to the G-protein-coupled receptor (GPCR) family and transiently activate GTP-binding proteins that remodel actin structures of the cytoskeleton to control the contractile machinery of the cell and direct cell migration [mm]. Dynamic actin rearrangements control the formation of pseudopodia during cell migration toward a target as well as the formation of protrusions that surround harmful particles and pathogens before internalization within the phagosome during phagocytosis (5–7). Chemokine receptors are essential for phagocyte function as they trigger the rearrangement of actin-containing structures required for cell motility, which is at the core of developmental and immunological processes and tissue maintenance and remodeling (8–10). Likewise, chemokine receptor signaling contributes to the differentiation, proliferation, and polarization of phagocytes, which are determining factors in host-pathogen interactions, inflammatory responses, inflammation resolution, and wound healing (4–6, 11, 12).

Zebrafish are increasingly used as a model species to study development and disease owing to the accessibility of the early life stages (embryos and larvae) for genetic analyses, chemical screens, and intravital imaging (6, 13–17). These useful features of the zebrafish have been exploited to study the roles of phagocytes in models of infectious and inflammatory diseases and cancer. In this review, we will illustrate how the zebrafish model contributed to our understanding of the role of chemokine signaling axes in phagocyte biology and highlight its main contributions to the understanding of chemokine signaling axes in phagocytes by addressing relevant homologies and analogies between mammalian and zebrafish phagocyte function. We will focus on the two major structural subfamilies of chemokine receptors, CC and CXC, and on the migratory properties of macrophages and neutrophils in the context of development and disease. We will discuss the regulatory role of atypical chemokine receptors (ACKRs), in shaping chemokine gradients and how duplication of chemokine receptor genes in zebrafish allows assessing sub-functionalization or loss/gain of function events and the challenges that gene duplication poses. Finally, we will discuss the potential of zebrafish models to further our understanding of chemokine receptors in innate immunity and immune-related disease.

## Fundamentals of Chemokine Signaling and Regulation

Chemokines are small secretory and transmembrane cytokines that induce directed chemotaxis of macrophages and neutrophils through their specific receptors under pathological and homeostatic conditions (5, 7, 18). Chemokine receptors belong to the chordate-restricted class A of (rhodopsin-like) heptahelical G-protein coupled receptors (GPCRs), which is grouped into four subclasses according to the pattern of highly conserved cysteine residues they display near their N-terminus (CC, CXC, CX3C, and XC) (5, 19). The cysteine motif of a chemokine receptor is followed by an “R” for “receptor” or an “L” for ligands and a number indicating the chronological order in which the molecules were identified (5, 19, 20). A further subfamily containing the characteristic motif CX has been identified only in zebrafish at present (19). Following nomenclature conventions, human chemokine receptors are written in capital letters, while those of other species use the lowercase to simplify the distinction between species. The structure of chemokine receptors consists of an intracellular COOH terminus, an extracellular NH2 terminus, and seven transmembrane domains linked by three extracellular and three intracellular loops (5, 12) Chemokine receptors mediate leukocyte trafficking during cell migration processes such as infection, damage, development, cell proliferation and differentiation (21–24). GPCRs are the largest and most diverse family of membrane receptors in eukaryotes and the most common pharmaceutical target making chemokine receptors attractive targets to treat chronic inflammatory conditions (12, 25).

Inactive chemokine receptors are coupled to heterotrimeric G proteins. The Gα subunit is bound to GDP (guanosine diphosphate) in resting conditions and exchanges the GDP molecule for GTP (guanosine triphosphate) when the chemokine receptor binds a cognate ligand. The GTP-Gα subunit complex dissociates from the receptor and the Gβ-γ heterodimer, which triggers the canonical downstream signal pathways that ultimately result in the intracellular mobilization of Ca^+2^ and the rearrangement of cytoskeletal components required by the vesicle trafficking machinery and for cell migration (5, 26–28). Besides the conventional G protein-dependent signaling pathways, chemokine receptors can directly activate JAK/STAT (Janus kinase /Signal transducer and activator of transcription) signaling, a pathway shown to induce chemotaxis of progenitor germ cells (PGCs) in zebrafish (6, 29–31). Furthermore, chemokine receptors can also signal through β-arrestin to mediate the internalization and intracellular degradation of chemokines and chemokine receptors (12, 30, 32, 33).

Chemokine networks are highly promiscuous and redundant and can result in antagonistic and synergistic interactions since different signaling pathways share signal transducing elements. Due to its complex nature, chemokine signaling axes build up tangled networks that need tight spatio-temporal regulation to evoke specific responses (34). Some regulatory mechanisms of chemokine signaling include biased signaling, allosteric modulation of receptor activation, receptor internalization, receptor dimerization, ligand sequestration and ligand processing (5, 28, 35, 36). Furthermore, the function of conventional chemokine receptors can be fine-tuned by ACKRs. These atypical chemokine receptors constitute a structurally diverse group unified by their shared function of shaping chemokine gradients. ACKRs cannot signal in the canonical G protein-mediated fashion, but most of them can signal through β-arrestins and mediate chemokine degradation (33, 37). Several studies demonstrate that the ligand-scavenging function of AKCRs provides an important regulatory mechanism during cell migration and phagocyte recruitment (33, 37–39).

## Zebrafish as a Window to Chemokine Receptor Functions

The zebrafish model has been successfully used to study how chemokine signaling networks determine macrophage and neutrophil functions and to ascribe these receptors a role in immunity, inflammation, and cancer models (4, 13, 16, 22, 40–42). It is a powerful vertebrate model well-suited for non-invasive *in-vivo* imaging given its optical transparency at early embryonic and larval stages. Transgenic lines specifically labeling neutrophils and macrophages by linking fluorescent proteins to the *mpx* and *lyz promoters* for the former, and the *mpeg1.1* and *mfap4* promoters for the latter, allow us to visualize and track these phagocytes at a whole organism level. A wide variety of gene-editing methods like CRISPR-Cas9 and transitory gene knockdown (morpholinos) or RNA-based gene overexpression can be delivered by microinjecting eggs at the single-cell stage (16, 43). The zebrafish model is ideal to assess developmental processes and since over 80% of all human disease genes identified so far have at least one functional homolog in zebrafish, it serves as a powerful animal model for human diseases too (22, 43).

**Figure 1:**
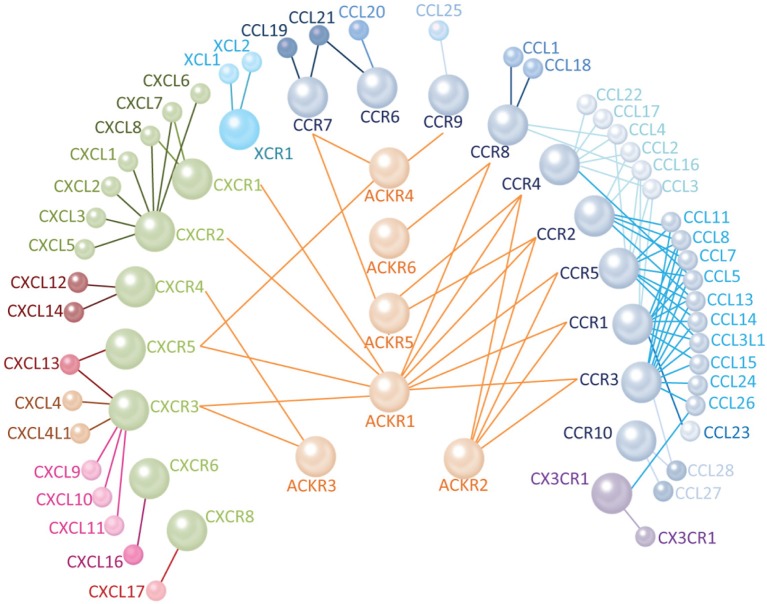
Human chemokine signaling networks are highly promiscuous. There are 25 receptors and 45 ligands in the human chemokine signaling network including seven members of the CXCR family (green), 1 XCR (cyan), 10 CCR (blue), and 1 CX3CR (violet). The CXCL chemokines are shown in shades of pink, XCL in cyan, CCL in shades of blue, and CX3CL in violet. The color intensity of the lines connecting receptors and ligands indicates the binding specificity. Darker colors indicate a higher binding affinity. There are six characterized AKCRs (orange) that antagonize the function of conventional chemokine receptors (connected with lines) by binding one or more of their ligands.

Most human chemokine receptors and ACKRs have at least one (putative) zebrafish ortholog (6, 30, 44) as shown in Table 1. The last common ancestor of humans and zebrafish went through two rounds of whole-genome duplication during vertebrate evolution (19). Subsequently, a series of intrachromosomal duplication events occurred in the taxon that led to zebrafish (4, 19, 44, 46). These events resulted in the duplication of several chemokine receptor genes that either preserved their original function, lost their function, or acquired a new one (19, 44). While most of the human chemokine receptor genes can be found as single or multi-copy genes in the zebrafish genomes, some cases remain unresolved (Figure 1). For example, no homologs of CCR1, CCR3, and CCR5 are currently annotated in the Zebrafish Information Network (ZFIN) database. Moreover, there are zebrafish chemokine receptors annotated without a human counterpart, such as Ccr11 and Ccr12. Also, a CX family of chemokine receptors has been identified that is restricted to (zebra) fish (6, 19, 44).

**Table 1:** Chemokine receptor genes, their ligands and their role in embryonic development, cancer progression, wound-induced inflammation and pathogen-driven inflammation.

**Chemokine receptor**	**Human**	**Ligands**	**Zebrafish**	**Ligands**	**embryonic development**	**Cancer progression**	**Wound-induced inflammation**	**Pathogen-driven inflammation**
CXCR1 (IL8RA)	CXCR1	CXCL6, 8 (IL-8)	Cxcr1 (Il8ra)	Cxcl8a (Cxcl8L1) Cxcl8b1, 3 (Cxcl8L2.1,0.3)		Neutrophil recruitment (15, 45). Sustained inflammation (15, 45–48). Tumor growth (45–47, 49). Tumor expansion (45, 47, 49).	Neutrophil recruitment, pro-inflammatory function (45, 47)	
CXCR2 (IL8RB)	CXCR2	CXCL1 (NAP3), 2 (MIP2 alpha), 3 (MIP2 beta), 5, 6, 7 (PPBP), 8 (IL-8)	Cxcr2 (Il8rb)	Cxcl8a (Cxcl8L1) Cxcl8b.1,0.2.3 (Cxcl8L2.1–0.3) Cxcl18b		Chronic inflammation (45, 47, 49).	Neutrophil reverse migration, anti-inflammatory function (45, 50, 51).	Neutrophil recruitment and bacterial clearance (51–55)
CXCR3	CXCR3A CXCR3B	CXCL4-B (PF4-B), 9-A/B (MIG-A/B), 10-A/B (IP-10A/B) 11A/B (I-TAC-A/B)	Cxcr3.1,2, 3	Cxcl11-like chemokines aa, ac, ad, ae, af and ag		Cell proliferation Cell survival Tumor expansion Angiostatic effect	Cxcr3.2 recruits macrophages and neutrophils to injury (47, 50, 56, 57). Cxcl11aa is a pro-inflammatory marker (M1) (58, 59).	**Cxcr3.2:** macrophage recruitment and motility (50, 56, 57), neutrophil recruitment (56, 57). **Cxcr3.3:** ligand scavenger, a regulator of Cxcr3.2 function (50).
CXCR4 (fusin)	CXCR4	CXCL12 (SDF1)	Cxcr4a Cxcr4b	Cxcl12b Cxcl12a	**Cxcr4a:** guidance of multicellular vessel growth and coordination of gastrulation movements (60, 61). **Cxcr4b:** progenitor germ cells (PGCs) (6, 31, 62–66).	Macrophage and neutrophil recruitment (45, 46, 67, 68). Tumor angiogenesis Tumor dissemination (67, 68).	Neutrophil recruitment and retention at the wounding site. Pro-inflammatory (69).	Neutrophil recruitment Bacterial clearance (55). Granuloma vascularization (52).
CCR2	CCR2	CCL2 (MCP1)	Ccr2	Ccl2 (mcp1)			Macrophage recruitment (53, 70). Ccr2 is an anti-inflammatory marker (M2) (71, 72).	Recruitment of permissive macrophages (71, 72).
ACKR3 (CXCR7)	ACKR3	CXCL11 (I-TAC) CXCL12 (SDF1)	Ackr3b (Cxcr7a/b)	Cxcl12a	Scavenges Cxcl12a to shape chemokine gradients (6, 36, 65, 66, 73).	Tumor angiogenesis Chemotaxis (74).		

This review will focus on the zebrafish homologs of human of CXCR1/2, CXCR3, CXCR4, ACKR3, and CCR2 (Supplementary Table 1) since these receptors have a known function in phagocyte function during development and inflammatory processes. Below we discuss how the genes encoding these receptors are conserved, and in some cases, duplicated in zebrafish. In the subsequent sections, we review how studies in zebrafish contributed to understanding the roles of these receptors in developmental and disease processes.

### The Cxcr1/2-Cxcl8 Signaling Axis

The CXCR1/2-CXCL8 signaling axis is one of the primary chemotactic pathways in neutrophils and of major interest to assess inflammatory processes (45). Zebrafish chemokine receptors Cxcr1 (Il8ra) and Cxcr2 (Il8rb) are functionally homologous to their mammalian counterparts. Furthermore, chemokines of the CXCL8 (IL-8) family, which interact with these receptors, are conserved between humans and zebrafish, while not present in mice (75). Cxcr1 and 2 are highly expressed on zebrafish neutrophils and mediate their recruitment by binding to their shared ligands Cxcl8a, Cxcl8b1, Cxcl8b2, and Cxcl8b3 (Cxcl8L2.1, 0.2, and 0.3, respectively) (6, 19, 47, 52). Cxcl8a and the three Cxcl8b variants are all reported to act via Cxcr1 and Cxcr2 to induce neutrophil recruitment, whereby no specific binding patterns involving the three Cxcl8b variants have been reported so far (6, 47). The Cxcl18b chemokine found in zebrafish and other teleost fish also attracts neutrophils via Cxcr2 (56). Whether this chemokine activates Cxcr1 remains unknown.

### The Cxcr3-Cxcl11 Signaling Axis

Human CXCR3 is predominantly expressed on T cells, but also multiple other leukocyte cell types, including macrophages (57, 58). The *cxcr3* gene is triplicated in zebrafish and the copies are referred to as *cxcr3.1, cxcr3.2*, and *cxcr3.3*. In humans, CXCR3 binds to CXCL9 (MIG: monokine induced by gamma interferon), CXCL10 (IP-10: interferon-gamma induced protein 10) and CXCL11 (I-TAC: inflammatory-inducible T-cell alpha chemoattractant) (19, 50). These chemokines are thought to be derived from a common *CXCL11*-like ancestral gene. In zebrafish seven *cxcl11*-like chemokine genes have been identified and are annotated as *cxcl11aa, ac, ad, ae, af*, and *ag* (57). The Cxcl11aa ligand has been functionally studied and was shown to mediate cell recruitment through Cxcr3.2 (48, 57, 58). Studies in zebrafish larvae have focused on *cxcr3.2* and *cxcr3.3*, which are expressed on macrophages and neutrophils while *cxcr3.1* is not detectable at this stage (57). While Cxcr3.2 appears to function as a conventional chemokine receptor, like human CXCR3, Cxcr3.3 has features of ACKRs such as a DCY motif instead of the highly conserved DRY motif that prevents classic G protein-mediated signaling (12, 48). Supporting that Cxcr3.3 regulates Cxcr3.2 function, these paralogs have antagonistic effects on macrophage recruitment to sites of infection and injury in zebrafish (48, 57). The functional antagonism between the zebrafish paralogs *cxcr3.2* and *cxcr3.3* can be viewed as a regulatory mechanism analogous to the functional antagonism of human *CXCR3* splice variants *A* and *B* (50, 62, 76, 77).

### The Cxcr4a/b-Ackr3/Cxcl12-Signaling Axis

CXCR4 signaling mediates functions of a variety of cell types, within and beyond the immune system (63, 78). The CXCR4-CXCL12 (SDF1: stromal cell-derived factor) axis is remarkably conserved between zebrafish and humans although both the receptor and ligand genes are duplicated in zebrafish and annotated as *cxcr4a/b* and *cxcl12a/b*, respectively (6, 30, 64). Both Cxcr4 receptors can bind both ligands, although Cxcr4a preferentially binds to Cxcl12b and Cxcr4b binds Cxcl12a with a higher affinity (29). The duplication of the *cxcr4* gene in zebrafish is a representative example of gene sub-functionalization. Cxcr4a is primarily associated with cell proliferation and vessel extension, while Cxcr4b regulates neutrophil and macrophage interactions with other cell types and has been implicated in the modulation of inflammation, neutrophil and macrophage migration, metastatic and angiogenic events, and tissue regeneration (29, 64, 65, 79). In mammals, CXCR4-CXCL12 is subject to modulation by an atypical chemokine receptor ACKR3, which binds the CXCR4 ligand CXCL12 but also the CXCR3 ligand CXCL11 (63, 66). The zebrafish *ackr3b (cxcr7b)* gene is on the same chromosome as *cxcr4a/b* and it has been shown that the Ackr3b protein binds both Cxcl12 and Cxcl11 but cannot induce cell migration (79–82). By competing with Cxcr4b for the shared Cxcl12a ligand, Ackr3b helps to maintain chemokine gradients during chemotaxis (79, 80). The potential interaction between Ackr3b and Cxcr3.2-Cxcl11aa signaling has not been characterized yet (70). As discussed below, Ackr3b has been implicated in several pathological conditions as well as in zebrafish development (6, 66, 79, 80, 82).

### The Ccr2-Ccl2 Signaling Axis

CCR2 is the receptor for monocyte chemoattractant protein −1 (MCP-1/CCL2) (53). Identifying zebrafish orthologs of human CC chemokine receptors has been challenging since multiple zebrafish *cc-* receptor genes have a remarkably high similarity to a single human *CC* chemokine receptor gene. However, a zebrafish *ccr2* orthologue could be identified in zebrafish, supported by functional evidence, as human CCL2 was shown to trigger macrophage recruitment in zebrafish embryos in a *ccr2*-dependent manner (53, 83).

The duplication of several chemokine receptor genes in zebrafish poses a challenge for the identification of homologies and at the same time, it provides an experimental platform to assess both loss of function and sub-functionalization events to further our understanding of chemokine signaling in phagocyte function as exemplified by the Cxcr4 and Cxcr3 paralogs (29, 48). In the following sections, we will illustrate how zebrafish embryonic development helped to unravel fundamental chemokine signaling mechanisms and discuss in detail the roles of zebrafish chemokine receptors Cxcr1/2, Cxcr3.2/3.3, Cxcr4b, Ackr3b, and Ccr2 in macrophage and neutrophil biology in the context of cancer and wound and pathogen-driven inflammation.

## Dissecting Chemokine Signaling Principles Using Developing Zebrafish

The chemokine signaling axes involved in phagocyte biology are also functional in other cell types of the developing zebrafish embryo (84). This model brought fundamental new insight into the principles of chemokine signaling. It was a long-held idea that the membrane-spanning domains and the extracellular portions of a chemokine receptor conferred signal specificity (85). However, recent work on zebrafish showed that cell identity and chemokine receptor signal interpretation modules (CRIM) are the major determinants for the functional specificity of a chemokine receptor-ligand interaction (84, 85). The directed expression of chemokine receptors that were not naturally expressed by a cell through mRNA injections of zebrafish eggs showed that the foreign receptor could overtake the function of the original receptor in the presence of its ligand. Even receptors that do not share high sequence similarities, like CC and CXC receptors, were found to evoke the same response if expressed on the same cell-type showing that CRIM process a generic signal into a discrete response that is dictated by the cell type. Consistent with the fact that cell identity and CRIM determine the functional specificity of chemokine receptors, the same chemokine receptor can elicit very different biological responses depending on the cell that expresses it (84). For example, when Cxcr4a is expressed on hematopoietic progenitor cells, it modulates chemotaxis, yet in neuronal progenitor cells, it inhibits proliferation (86).

Studies in zebrafish embryos also contributed to elucidate regulatory mechanisms of chemokine signaling. One such process is the cleavage of certain chemokines (like Cxcl8) by matrix metalloproteinases (MMPs) to activate and confer them enhanced chemotactic properties. The use of a broad-spectrum MMP inhibitor showed reduced neutrophil and macrophage recruitment to sterile heart injury in zebrafish showing that MMPs are key mediators of inflammation and tissue regeneration (36). An outstanding example of ACKR-mediated regulation of chemotaxis comes from the characterization of the paralogs *cxcr4a* and *cxcr4b* and the interaction of the latter with Ackr3b to fine-tune single-cell migration during development. The Cxcl12b-scavenging function of Ackr3 is required for shaping a self-generated chemokine gradient that guides the migration of the lateral line cell primordium (6, 60, 79, 80). An analogous Cxcr4/Ackr3/Cxcl12 system indispensable to form an endogenous chemokine gradient within the mouse lymph node was described later, confirming the observation made in zebrafish (61). In fact, the identity of Ackr3b as a scavenger receptor that signals via β-arrestins was first described in zebrafish and later confirmed in human cells and mice (38). Similarly, Cxcr1/2-Cxcl8 driven migration of neutrophils along immobilized gradients within tissue was first described in zebrafish (73). During this process, tissue-bound chemokine gradients form through the binding of chemokine and heparan sulfate proteoglycans (HSPGs) resulting in a process called haptotaxis. This type of cell movement coordinates both directional guidance of cells (orthotaxis) and motility restriction in the proximity of the source of the chemotactic signal (73). Haptotaxis was later confirmed in murine dendritic cell recruitment via Ccl21 (87).

Among the chemokine receptors of phagocytes, it is especially the interacting Cxcr4/Ackr3 pair that has much broader roles in developmental processes. We briefly summarize the zebrafish studies that revealed these developmental roles below, which are important to take into account also when studying immune cell functions.

### The Cxcr4a/b-Ackr3-Cxcl12 Axis in Development

Cxcr4a is mainly involved in guiding multicellular vessel growth (88) and in controlling proper gastrulation movements by ensuring adhesion between cell-matrix and endodermal cells (49). The Cxcr4b-Cxcl12a signaling axis regulates the migration of a wide range of cell types including neuronal cells, axons, neutrophils, neural crest cells, endothelial cells, and muscle cell precursors (6, 33, 49, 80, 88). Primordial germ cells express Cxcr4b and migrate toward Cxcl12a gradients tracing their migration route. These cells specifically respond to Cxcl12a and neglect the Cxcl12b ligand, involved in other developmental processes, which can be found along their migration path. Ackr3b, expressed mostly by somatic cells, plays a fundamental role in removing Cxcl12b from the extracellular space and clearing the path for PGC migration (31, 63, 65, 78). It scavenges chemokines to shape time and tissue-specific gradients to tightly regulate developmental processes involving cell migration (6, 79, 80). The Cxcr4a/b- Ackr3-Cxcl12 interaction was first observed *in vivo* during zebrafish PGCs migration (33). Ackr3 orchestrates the lysosomal degradation of Cxcl12a in a β-arrestin-dependent process while the receptor itself is recycled back to the plasma membrane (37). Moreover, the scavenging activity of Ackr3b is crucial for the maintenance of a self-generated chemokine gradient that directs the migration of the lateral line primordium during the development of the zebrafish posterior lateral line (PLL) (60, 79, 80).

## Chemokine Receptors in Cancer Progression

Cancer progression is strongly influenced by chemokine-dependent leukocyte recruitment and infiltration into primary tumors as well as by the subsequent dissemination of cancer cells from primary tumors into adjacent and distant tissues (15, 76, 89). Live visualization of fluorescently labeled tumor cells in zebrafish larvae enables early assessment of vascular remodeling events, tumor dissemination, and metastasis at the organismal level (24, 64). Zebrafish cancer models are also suitable to image early tumor-initiation events and the crucial interplay between the tumor cells and the microenvironment (45). In particular, xenotransplantation models, in which human invasive cells are systemically inoculated into zebrafish larvae, are useful to assess the interactions between human tumor cells and host leukocytes that underlie early metastatic onset (67). Additionally, the larval zebrafish system offers a simple and robust screening platform for anti-tumor compounds targeting different stages (angiogenesis, metastasis, etc.), further emphasizing its translational value (24, 64).

The tumor environment is a highly inflammatory focus that attracts leukocytes through secretion of cytokines of different natures, including chemokines (45). Chemokine receptors CXCR1, 2, 3, 4, and 7 have been implicated in tumor angiogenesis, sustaining tumor growth and expansion both in zebrafish and humans, as discussed below. The role of CCR chemokine receptors in cancer using the zebrafish model has not been addressed yet.

### The Cxcr1/Cxcr2-Cxcl8 Axis in Cancer

Neutrophils are the first responders to acute inflammation, infection, and damage. These cells exhibit remarkable phenotypic plasticity that is determined by the integration of extracellular cues (45). In zebrafish, cancer cells recruit neutrophils through chemokine receptors Cxcr1 and 2 and their Cxcl8 ligands (15, 75). Neutrophil populations have a dual role in the development of different cancers. Tumor-associated neutrophils (TANs) directly engage with tumor cells and are reported to support tumor growth, tissue invasion and angiogenesis mimicking sites of chronic inflammation. In contrast, anti-tumor neutrophils undergo apoptosis and reverse migration back into the vasculature, thereby favoring the resolution of inflammation (45, 75). Using the zebrafish model, it became clear that TANs are recruited to tumor-initiating sites through the Cxcr1-Cxcl8a pathway and that in this context, Cxcr2 is not required for efficient neutrophil recruitment. Fewer neutrophils are recruited to tumor-initiating foci in *cxcr1* mutant zebrafish larvae and proliferation of tumor cells is restricted, suggesting that TANs are critical for early stages of neoplasia and tumorigenesis (75). In agreement with these observations, Cxcr1 expression is lower in anti-tumor neutrophils that display a predominantly anti-inflammatory phenotype (52, 68).

### The Cxcr4a/b-Ackr3–Cxcl12 Axis in Cancer

A vast body of literature associates the chemokine receptor CXCR4 with the development of cancer pathogenesis in humans, mice and zebrafish (6, 15, 24, 50, 74). Cxcr4b is highly expressed on zebrafish neutrophils and together with its ligand Cxcl2a, it facilitates tumor angiogenesis and dissemination into different tissues by attracting malignant Cxcr4-expressing cells into healthy organs and tissues where ligand can be found (63, 74, 76). Zebrafish larvae lacking *cxcr4b* (*ody* mutants) fail to induce micrometastases and to sustain human cancer cells after xenotransplantation. Basal neutrophil motility is attenuated and whole-body neutrophil counts are lower in *cxcr4b* mutants than in wild type (wt) larvae (67). Accordingly, tumors in *cxcl12a* mutant zebrafish cannot metastasize, further supporting that Cxcr4b signaling promotes tumor expansion (64).

While neutrophils are important cellular mediators of inflammation and play a central role in tumor initiation and expansion macrophages represent a significant amount of the leukocytes that infiltrate tumors. Macrophages phagocytose cancer cells and dying neutrophils whilst secreting immunomodulatory cytokines. Macrophages also express Cxcr4b and respond to Cxcl12a (11, 90). A study focused on glioblastoma progression used the zebrafish model to show that tumor cells secrete Cxcl12a to recruit macrophages to the tumor site (90). Cxcr4b-Cxcl12a signaling in macrophages is also linked to tumor-promoting functions by enhancing proliferation and invasiveness, modifying the extracellular matrix and favoring tumor neovascularization (15, 28, 65). Interestingly, live visualization of zebrafish macrophages and microglia showed dynamic interactions with cancer cells which did not result in phagocytosis of the malignant cells, thereby avoiding an anti-tumor function of macrophages (67). *cxcr4b* mutant larvae had a lower tumor burden in this context too and depletion of macrophages and microglia significantly reduced oncogenic cell proliferation, suggesting that Cxcr4b signaling promotes macrophage infiltration during initial stages of brain cancer (90).

As discussed above, Cxcr4b signaling can be fine-tuned through ligand scavenging by the atypical Ackr3b receptor. Human ACKR3is linked to tumor growth, invasion, and metastasis (11). Tumor cells and vascular endothelial cells of different tissues show an increased expression of Ackr3 and it has been suggested to include this receptor as a marker for cancer (63). A study by van Rechem et al. (91) found that Ackr3 is a direct target of the tumor suppressor HIC1 (Hypermethylated in Cancer 1) which is inactive in many human tumors. The role of Ackr3b in cancer pathogenesis is still unknown in zebrafish and as multiple studies found that Ackr3b depletion results in severe developmental abnormalities (6, 29, 30, 37), a gene knockout/down approach to assessing its role in cancer progression would require the development of cell-specific or conditional knockout systems.

## Chemokine Receptors in Wound-Induced Inflammation

The zebrafish model is well-suited to assess aseptic wound-induced inflammation and tissue regeneration either by amputating the ventral or tail fin or by pinching tissue with sterile needles (68, 69, 92). Recruitment of neutrophils first, and macrophages in a later phase, is key during the inflammatory response, which is broadly divided into three phases: early leukocyte recruitment, amplification or acute inflammation, and resolution (69). Neutrophils recruited shortly after damage secrete chemokines that activate tissue-resident cells and recruit more leukocytes to the injury, thereby amplifying inflammation. As described in the previous section, Cxcl8a is a strong neutrophil attractant and therefore, a central element at all stages of the inflammatory process (68, 69, 71). Neutrophils are known to be short-lived and to undergo apoptosis shortly after activation (40). However, a recently characterized subpopulation of neutrophils that returns to the circulation after activation has a longer lifespan and an anti-inflammatory effect (68, 69). The tail-amputation model using larval zebrafish is well-suited for tracking neutrophil reverse migration since it enables *in-vivo* tracking of these cells at different stages of the inflammatory response (72, 92). It helped to establish that neutrophils recruited upon injury emerge from hematopoietic tissue in the proximity of the affected area, that they shuttle between the vasculature and the injury during acute inflammation and redistribute in a proximal direction to different sites of the body during the resolution phase (72). A detailed assessment of the transition from neutrophil recruitment and clustering during acute inflammation and neutrophil redistribution during the resolution phase showed to be regulated through Cxcl8a-induced trafficking and turnover of Cxcr1 and Cxcr2 on the membrane of neutrophils (71).

Two distinct subtypes of macrophages, pro-inflammatory and anti-inflammatory, drive the formation of a mass of highly proliferative stromal cells called blastema and subsequent tissue remodeling during epimorphic regeneration (51, 93). Using the zebrafish tail-amputation model with fluorescently labeled macrophages (mCherry) and Tnfa (GFP), Nguyen-Chi et al. showed that shortly after tail amputation both pro-inflammatory (GFP+) and ant-inflammatory macrophages (GFP-) accumulated in damaged tissue and that anti-inflammatory macrophages remained associated to the injury until regeneration was completed unlike pro-inflammatory macrophages, which retracted from the area. Chemical depletion of macrophages showed that the initial interaction between TNFa-expressing macrophages and the damaged area is required for blastema formation. Knockdown of the Tnfa receptor *tnfar1* confirmed that Tnfa is fundamental for fin regeneration as it primes blastema cells to undergo regeneration in zebrafish (93). This phenotypic polarization dynamics in macrophages had been reported in cell culture but it had not been confirmed in a live system. Below we discuss the chemokine receptors implicated in the wound-induced macrophage and neutrophil migration and polarization responses.

### The Cxcr1/2-Cxcl8 Axis in Wound-Induced Inflammation

Both Cxcr1 and Cxcr2 are required for efficient recruitment of neutrophils to damaged areas at the initial stage of the inflammatory response (52). Cxcr2 and Cxcl8a (Cxcl8L1) and Cxcl8b (Cxcl8L2) are transcriptionally upregulated after tail amputation in zebrafish. However, Cxcl8a and Cxcl8b have differential roles in neutrophil migration during inflammatory responses. Cxcl8a mainly orchestrates neutrophil recruitment to sites on injury whereas Cxcl8b redirects neutrophils back into the bloodstream (94). Work in zebrafish also showed that the bidirectional movement of neutrophils between the injury and vasculature during acute inflammation is coordinated by distinct roles of Cxcr1 and Cxcr2 (75, 95). Neutrophils that undergo reverse migration express lower levels of Cxcr1 relative to Cxcr2, suggesting that Cxcr2 is involved in recruiting neutrophils back into the vasculature. Further research showed that the Cxcr1-Cxcl8a axis recruits neutrophils to the inflammatory focus while Cxcr2-Cxcl8a orchestrates reverse migration and resolution of inflammation (89). Recently, Coombs et al. showed that both Cxcr1 and Cxcr2 mediate the initial recruitment of neutrophils to damaged tissue but that these receptors exert different functions during the transition from acute inflammation to the resolution phase. Cxcr1 shows a strong initial response toward Cxcl8a but undergoes gradual desensitization followed by receptor internalization, whereas Cxcr2 remains stably expressed on the plasma membrane with sustained responsiveness toward Cxcl8b, and orchestrates neutrophil dispersal during the resolution phase (71).

### Cxcr3 and Ccr2 Axes in Wound-Induced Inflammation

Macrophages are crucial players of the inflammatory response triggered by tissue damage and exhibit remarkable phenotypic plasticity (51, 54). Live tracking of fluorescently labeled macrophages in zebrafish showed that these cells are recruited to injury shortly after neutrophils at early stages [several papers]. Cxcr3.2, a functional CXCR3 ortholog in zebrafish, and Ccr2 both mediate the recruitment of macrophages to injury (48, 53, 57, 58, 83). Mutation of *cxcr3.2* and knockdown of *ccr2* result in attenuated recruitment of macrophages to the wound (57, 58). Cxcr3.2 depletion also reduced neutrophil recruitment, unlike Ccr2 knockdown which affected macrophages only (48, 58, 83). At the beginning of the inflammatory response, macrophages acquire a pro-inflammatory phenotype characterized by the secretion of inflammatory markers (M1) like Tnfa, Il1-b, and the Cxcr3.2 ligand Cxcl11aa. As the inflammatory process develops, they transit toward an anti-inflammatory phenotype (M2) characterized by the expression of chemokine receptor Ccr2 and Cxcr4b (51). Ccr2 is thought to mediate the transition from acute inflammation [M1] to tissue regeneration processes [M2] as phagocytosis of necrotic and apoptotic neutrophils by macrophages is associated with the beginning of tissue regeneration (69, 93).

### The Cxcr4a/b-Ackr3-Cxcl12 Axis in Wound-Induced Inflammation

The chemokine signaling axis Cxcr4b-Cxcl12a is required for the proper development and distribution of neutrophils at early developmental stages and sustains inflammation by recruiting and retaining neutrophils at sites of injury (40, 96). CRISPR-Cas9-mediated knockdown of Cxcr4b and Cxcl12b significantly increased the clearance of apoptotic neutrophils by macrophages and enhanced reverse migration of neutrophils thereby ameliorating inflammation. Chemical inhibition of the Cxcr4b-Cxcl12a axis leads to a faster resolution of inflammation by hindering the retention of neutrophils at the inflammatory site (68, 97). Dominant gain-of-function truncations of CXCR4 are associated with warts, hypo-gammaglobulinemia, infections, and myelokathexis (WHIM) syndrome, a primary immunodeficiency disorder characterized by neutropenia (96). The expression of homologous Cxcr4 WHIM truncations in zebrafish showed that neutrophil release into the blood was impaired and recruitment to injury after fin amputation was diminished. Larvae with the WHIM-truncated Cxcr4b displayed aberrant neutrophil development and distribution due to reduced chemotaxis, which could be reverted upon Cxcl12a depletion, suggesting that WHIM truncation increases Cxcr4b sensitivity toward Cxcl12a (96).

The possible interaction between Cxcr4b and Ackr3b during inflammation has not yet been addressed.

## Chemokine Receptors in Pathogen-induced Inflammation

Chemokine receptors play a fundamental role in the immune response against invading pathogens by mediating leukocyte trafficking to sites of infection (3, 4, 98). Bacterial infections can be followed from very early stages and with great detail using cell-specific fluorescent transgenic zebrafish lines and fluorescent bacteria. The optically clear larvae facilitate live visualization of complex host-pathogen interactions at the whole organism level and at the same time, it provides a reasonably simplified setting to assess chemokine signaling when used before adaptive immunity develops (55, 96, 98). Most of the studies on chemokine receptor function in the context of infection were performed with the zebrafish-*Mycobacterium marinum* (*Mm*) model for tuberculosis. This model provides a surrogate system that strongly resembles *Mycobacterium tuberculosis* (*Mtb*) pathogenesis in humans, including the formation of granulomas, the histological hallmark of tuberculosis. *Mm* is a natural pathogen of teleost fish and a close genetic relative of *Mtb* which permits assessing co-evolution between host and pathogen (55). Both *Mm* and *Mtb* can survive intracellularly in macrophages. Macrophages are the primary components of granulomas and play a dual role in mycobacterial pathogenesis. Macrophage recruitment to infection sites is crucial for neutralizing mycobacteria but it also provides them with a niche for replication and a vector for dissemination into host tissues (59).

### The Cxcr2-Cxcl8 Axis in Pathogen-Induced Inflammation

Cxcr2 (but not Cxcr1) mediates infection-induced neutrophil mobilization from the caudal hematopoietic tissue (CHT) to infectious foci (99). Neutrophils are very efficient at killing pathogens through degranulation and the rapid release of reactive oxygen species (ROS) (100). Mycobacteria primarily infect macrophages to replicate and expand at initial stages of infection (83). At later stages, when the infection is well-established, neutrophils are recruited primarily through Cxcr2 and Cxcl8a secreted by macrophages and epithelial cells (101, 102). Unlike Cxcl8a, Cxcl18b is secreted by non-phagocytic cells of the stroma within granulomatous lesions during *Mm* infection (56). Neutrophils contribute to the phagocytosis and destruction of infected macrophages and are therefore crucial to control mycobacterial infection (101, 103).

### The Cxcr3-Cxcl11 and Ccr2-Ccl2 Signaling Axis in Pathogen-Induced Inflammation

Chemokine receptors direct the course of mycobacterial infection by controlling leukocyte recruitment with distinctive microbicidal properties (51, 53, 93). *Mm* recruits macrophages at the early stages of infection through the Cxcr3.2 and Ccr2 chemokine receptors (48, 57, 83). Cambier et al. (83) proposed that phenolic glycolipid in the bacterial cell wall induces ccl2 transcription and recruits blood circulating monocytes via Ccr2 in a toll-like receptor-independent way. The monocytes recruited via Ccr2 are permissive to mycobacterial replication and are less efficient clearing the pathogen because they contain less inducible nitric oxide synthases (83). On the other hand, the authors suggest that toll-like receptor-mediated recruitment of tissue-resident macrophages primes cells to adopt a microbicidal phenotype and that mycobacteria evolved different mechanisms to evade detection by these cells. Once Ccr2-expressing monocytes are recruited, mycobacteria can transfer from the microbicidal tissue-resident macrophages to the Ccr2-expressing permissive monocytes. This permissive monocyte recruitment driven by mycobacteria will amplify the infection as infected macrophages that egress from the granuloma seed secondary granulomas away from the initial infection site (53). Interestingly, Cxcl11aa (the main ligand of Cxcr3.2) is induced in a manner dependent on the myeloid differentiation response gene 88 (myd88) (104). Myd88 serves as an adaptor molecule for the majority of toll-like receptors suggesting that macrophages recruited through Cxcr3.2 might have different microbicidal properties than those recruited through Ccr2 (104, 105).

**Figure 2:**
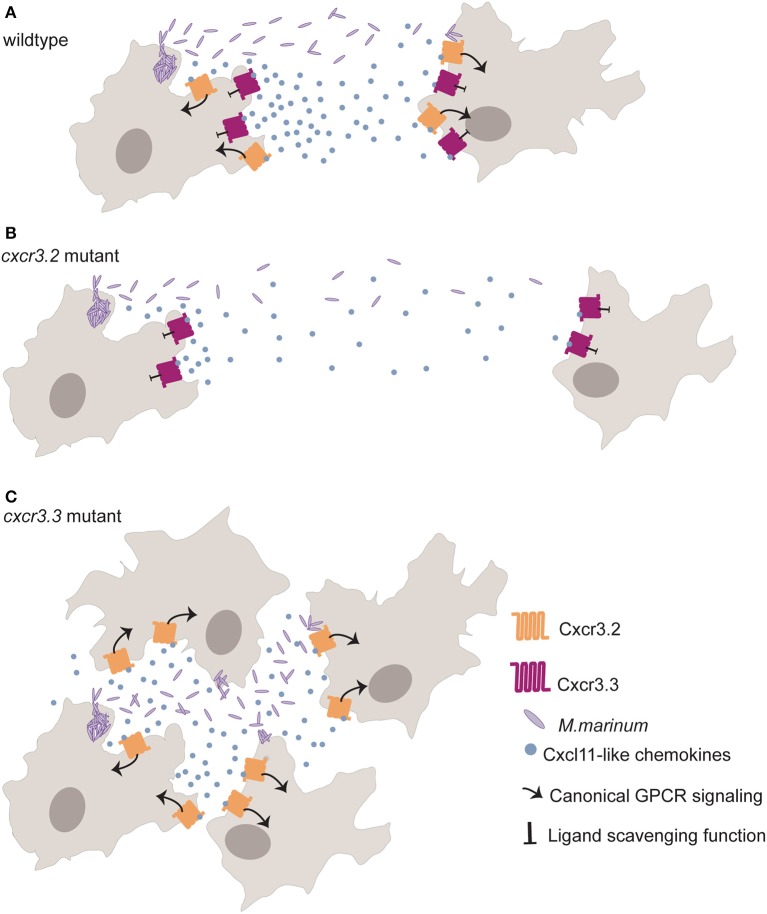
The paralogs *cxcr3.2* and *cxcr3.3* have antagonistic functions that regulate macrophage recruitment to sites of infection. Cxcr3.2 (orange) is a functional homolog of human Cxcr3 required for macrophage recruitment to sites of infection and other inflammatory settings. Cxcr3.3 (dark red) displays the structural of Ackrs such as the substitution of the central Arginine (R) of the highly conserved E/DRY-motif for a Cysteine (DCY) that prevents canonical GPCR signaling (arrow). Cxcr3.3 regulates Cxcr3.2-mediated macrophage recruitment through its scavenging function (blunt arrow) of Cxcl11-like chemokines (blue dots). **(A)** Shows how macrophages infected with *M. marinum* (purple rods) recruit non-infected macrophages through the secretion of Cxcl11-like chemokines to contain the bacterial infection and to clear dying macrophages in wt zebrafish larvae. **(B)** shows how macrophage recruitment is reduced in *cxcr3.2* mutants (as an actively signaling chemokine is depleted) and how fewer macrophages become infected with *M. marinum* due to reduced macrophage motility, favoring the contention of mycobacterial infection. **(C)** shows enhanced recruitment of macrophages to sites of infection due to an exacerbated Cxcr3.2 signaling because of higher ligand availability in absence of the scavenging function of Cxcr3.3. The dissemination of mycobacteria into these newly recruited macrophages will later seed secondary granulomas, supporting the dissemination of the infection.

The depletion of either Ccr2 or Cxcr3.2 results in a reduced recruitment of macrophages to sites of infection (53, 57, 58). However, *cxcr3.2* knockout limits *Mm* dissemination as fewer macrophages are recruited to sites of infection due to aberrant macrophage motility that prevents macrophage-mediated seeding of secondary infectious foci (57). Cxcr3.3 restricts Cxcr3.2 function in macrophages through its Cxcl11aa-scavenging function. Macrophages of *cxcr3.3* mutant zebrafish larvae are more mobile than wt controls, and recruitment to sites of infection and injury is, therefore, more efficient. Cxcr3.3 depleted larvae, show exacerbated Cxcr3.2 signaling due to higher ligand bioavailability and enhanced bacterial dissemination resulting from higher macrophage motility (48) (Figure 2).

### The Cxcr4a/b-Ackr3-Cxcl12 Axis in Pathogen-Induced Inflammation

As mentioned in previous sections, neutrophils are recruited through Cxcr4b and the chemokine Cxcl12a (68, 97). The depletion of Cxcr4b in zebrafish led to a significant reduction in neutrophil recruitment to infectious foci and a higher bacterial burden further emphasizing the relevance of neutrophils in the control of mycobacterial infection (101). Macrophages expressing Cxcr4b have been implicated in the delivery proangiogenic signaling within the granulomatous structures although the mechanism is unknown. Granulomas in *cxcr4b* depleted zebrafish larvae were poorly vascularized, bacterial growth was restricted and dissemination reduced (106).

## Concluding Remarks

The zebrafish model significantly contributed to the expansion of our knowledge on phagocyte behavior, function, and properties in the context of development, cancer progression, and sterile and pathogen-driven inflammation. Due to its genetic accessibility, zebrafish can be exploited to model congenital syndromes involving chemokine receptors implicated in leukocyte function, such as the WHIM syndrome (96). It has been of great value to unveil fundamental principles underlying chemokine signaling regulation, signal integration and to explore receptor sub-functionalization events (6, 17, 98). Furthermore, the functional diversification of duplicated chemokine receptor genes in zebrafish might reveal core mechanisms of chemokine signaling, like the ligand processing function of MMPs and the Cxcr3.2-Cxcr3.3 functional antagonism, and expand our knowledge on the function and interaction of ACKRs as well as to identify and explore analogous regulatory systems in humans (36, 48).

The tight connection between chemokine receptors and macrophage and neutrophil recruitment posits them as interesting therapeutic targets to treat chronic inflammation, a condition that can be induced by persistent infections like mycobacterial infections and precedes pathologies like cancer, autoimmune diseases and tissue damage (68, 69). The development of antibodies targeting chemokine receptors or chemokines that mediate neutrophil recruitment like Cxcr1/2-Cxcl8 and Cxcr4/ Ackr3-Cxcl12 could be used as an alternative anti-inflammatory and anti-oncogenic treatment to modulate neutrophil recruitment to inflammatory foci and tumor-initiating niches, respectively (75). Promoting neutrophil reverse migration to accelerate the resolution of inflammation by pharmacologically inhibiting Cxcr1-Cxcl8a signaling presents another approach to counteract inflammation and to restrict tumor progression (45, 97). While pharmaceutical targeting of the Cxcr4/ Ackr3-Cxcl12 signaling axis to inflammatory conditions remains plausible, it should be noted that this pathway is central for embryonic development and therefore, a developing organism like zebrafish larvae, might not be an optimal model for screening compounds targeting these axes (6, 30).

CXCR3 signaling in cancer also presents a therapeutic target. Unlike the mutation of *ackr3b, cxcr3.2* and *cxcr3.3* mutant larvae showed no major effects on embryonic development. Therefore, in future work zebrafish larvae can be used to screen chemical inhibitors targeting the CXCR3 axis. Studies show that disrupting CXCR3 signaling using chemical antagonists results in lower tumor burden in human lung cancer due to reduced cell proliferation and survival as well as increased caspase-independent cell death (107). However, CXCR3 has also been ascribed an angiostatic effect that blocks tumor neovascularization and some of its platelet-derived ligands work as anti-tumor agents by inhibiting lymphangiogenesis (108). The role of Cxcr3 and Cxcr4 signaling axes and their interaction with Ackr3b in cancer progression have not been explored using the zebrafish model in the context of cancer, but it could contribute to clarify the discrepant observations made so far. Also, the disruption of Cxcr3.2 signaling in mycobacterial infection resulted in reduced granuloma formation in zebrafish, similar to CXCR3 knockout in mice (109). Fine-tuning CXCR3 signaling could, therefore, serve the development of host-directed antibacterial therapies to circumvent the treatment limitations imposed by the ever-growing multi-drug resistance of bacterial strains.

Considering that chemokine receptors mediate interactions between macrophages and their extracellular environment, it would be interesting to unravel the chemotactic cues underlying macrophage polarization and their localization during infectious, inflammatory and tissue regeneration processes. Therapies aimed at enhancing macrophage efferocytosis (clearance of apoptotic cells by phagocytes) of neutrophils during inflammation or biasing macrophage polarization toward an anti-inflammatory and regenerative phenotype could serve as novel targets of regenerative drugs (93). Zebrafish stands out as a powerful model to study macrophage functional plasticity during inflammation in real-time and within a whole organism mostly because of the availability of several M1 transgenic lines. The generation of fluorescent transgenic zebrafish lines for M2 markers, such as *cxcr4b* and *ccr2*, would be helpful to further dissect the role of chemokine receptor signaling in macrophage polarization (51, 93). Fine-tuning macrophage polarization could enable us to prime macrophages to adopt an inflammatory phenotype that favors pathogen clearance or a tissue-regenerative phenotype to reduce inflammation as a therapy against multiple pathogens and conditions.

Due to its accessibility and its many advantages, the zebrafish model keeps up with state-of-the-art technologies, such as genome editing techniques like CRISPR/Cas9, the application of cell/tissue-specific RNA-sequencing and proteomics analyses (16, 43, 98). Together with cutting-edge microscopy techniques like super-resolution microscopy and lattice light-sheet microscopy, which can provide information about dynamic intracellular processes, the identity of chemokine receptors' downstream effectors and signal integration events can be further investigated. The link between chemokine signaling and relevant intracellular processes, like autophagy, in several contexts, could be assessed in homeostasis and disease to reveal fundamental signaling and physiological mechanisms within phagocytes.

## Author Contributions

FS and AM wrote the manuscript. VT made Figure 1 and reviewed the manuscript. All authors commented on the manuscript and approved the final version.

### Conflict of Interest

The authors declare that the research was conducted in the absence of any commercial or financial relationships that could be construed as a potential conflict of interest. The reviewer MS declared a past co-authorship with one of the authors, VT, to the handling editor.
