# Supplementary material for: Chemokine Receptors and Phagocyte Biology in Zebrafish
Source: Front Immunol. 2020 Feb 25;11:325. doi: 10.3389/fimmu.2020.00325 (PMC7053378; doi:10.3389/fimmu.2020.00325)
Supplement: Supplementary file 1 [file Table_1.docx]

**Supplementary table 1.**

Chemokine receptor genes and their accession numbers.

| Receptor | Accession number | Ligands | Accession number |
| --- | --- | --- | --- |
| Human | | | |
| *CXCR1* | ENSG00000163464 | CXCL6  CXCL8 | ENSG00000124875  ENSG00000169429 |
|  |  |  |  |
| *CXCR2* | ENSG0000018087 | CXCL1  CXCL2  CXCL3  CXCL5  CXCL7 | ENSG00000163739  ENSG00000081041  ENSG00000163734  ENSG00000163735  ENSG00000163736 |
|  |  |  |  |
|  |  |  |  |
|  |  |  |  |
|  |  |  |  |
|  |  |  |  |
| *CXCR3* | ENSG00000186810 | CXCL4  CXCL9  CXCL10  CXCL11 | ENSG00000109272  ENSG00000138755  ENSG00000169245  ENSG00000169248 |
|  |  |  |  |
|  |  |  |  |
|  |  |  |  |
| *CXCR4* | ENSG00000121966 | CXCL12 | ENSG00000107562 |
| *CXCR7 (ACKR3)* | ENSG00000144476 |  |  |
| *CCR2* | ENSG00000121807 | CCL2 | ENSG00000108691 |
|  |  |  |  |
| Zebrafish | | | |
|  |  |  |  |
| *cxcr1* | ENSDARG00000052088 | *cxcl8a*  *cxcl8b.1*  *cxcl8b.3* | ENSDARG00000104795  ENSDARG00000102299  ENSDARG00000099169 |
| *cxcr2* | ENSG00000180871 | *cxcl19*  *cxcl18b* | ENSDARG00000102776  ENSDARG00000075045 |
| *cxcr3.1*  *cxcr3.2*  *cxcr3.3* | ENSDARG00000078177 ENSDARG00000041041  ENSDARG00000070669 | *cxcl11-like chemokines aa,*  *ac,*  *ad,*  *ae,*  *af*  *ag*  *ah* | ENSDARG00000100662  ENSDARG00000092423  ENSDARG00000093779  ENSDARG00000116337  ENSDARG00000094706  ENSDARG00000113389  ENSDARG00000095747 |
| *cxcr4a*  *cxcr4b* | ENSDARG00000057633  ENSDARG00000041959 | *cxcl12a*  *cxcl12b* | ENSDARG00000037116  ENSDARG00000055100 |
| *cxcr7a*  *cxcr7b* | ENSDARG00000062478  ENSDARG00000058179 |  |  |
| *ccr2* | ENSDARG00000105363 | *ccl2* | ENSDARG00000098460 |
